# Editorial: Neurologic correlates of motor function in cerebral palsy: opportunities for targeted treatment, volume II

**DOI:** 10.3389/fnhum.2024.1525962

**Published:** 2024-12-02

**Authors:** Jessica Rose, Christos Papadelis

**Affiliations:** ^1^Division of Pediatric Orthopaedics, Stanford University School of Medicine, Stanford, CA, United States; ^2^Neuroscience Research, Jane and John Justin Institute for Mind Health, Cook Children's Health Care System, Fort Worth, TX, United States

**Keywords:** cerebral palsy, neurology, brain structure, mobility, gait, upper limb function

Cerebral palsy (CP) is the most common pediatric motor disorder, caused by injury to the developing brain. Three types of CP: spastic, dyskinetic and ataxic CP, arise from different regional brain injury and are characterized by different primary neuromuscular and secondary musculoskeletal impairments. Spastic CP, the most common type, arises from corticospinal tract (CST) injury, dyskinetic CP arises from basal ganglia and thalamus injury, and ataxic CP is associated with cerebellar vermis injury (Zhou et al., 2017). Current treatments for CP are only partially effective at improving neuromuscular impairments and mobility. Therefore, a better understanding of neuromuscular impairments and their impact on mobility can lead to more effective treatments. This Research Topic, “*Neurologic correlates of motor function in cerebral palsy: opportunities for targeted treatment, volume II*” highlights current research that investigates the underlying neuromuscular impairments of spastic CP, their impact on mobility, and promising emerging treatment.

Spastic CP is characterized by four interrelated neuromuscular impairments, two that result from reduced descending neural *activation*: weak muscle and reduced muscle growth relative to skeletal growth resulting in short muscle and joint contracture; as well as two neuromuscular impairments that result from reduced neural *inhibition*: muscle spasticity characterized by increased sensitivity to stretch, and impaired selective motor control characterized by flexion and extension synergies that interfere with voluntary movements (Zhou et al., 2017). Mirror movements also impact motor control and are thought to result from increased dependence on preserved motor pathways (Hruby et al., 2023).

While interventions for spastic CP often focus on tone, mobility is more often limited by weak and short muscle due to reduced neural activation and reduced muscle growth. Further, short muscles, especially two-joint muscles, are particularly vulnerable to spasticity, the increased sensitivity to stretch as the muscle extends over both joints. Pain, illness, inflammation and anxiety can be mistaken for “increased spasticity,” especially in individuals who have limited mobility and limited communication, highlighting the need for improved health monitoring and exercise. Misidentification of neuromuscular impairments such as misidentifying short muscle as muscle spasticity, leads to imprecise diagnosis, over treating muscle spasticity and under recognition of the impacts of sedentary life and reduced muscle growth on mobility and wellbeing.

Clinically, muscle growth is inadequately monitored for children with spastic CP and warrants focused clinical research. Current treatments for joint contracture, such as surgical tendon lengthening and skeletal realignment address musculoskeletal deformities that are years in the making, and therefore, have suboptimal outcomes. Early improved treatments for the underlying neuromuscular impairments of muscle weakness and reduced muscle growth could normalize musculoskeletal growth and prevent joint contracture and skeletal malalignment, minimizing the need for surgery. Muscle strength and length are highly responsive to stimuli (Greve et al., 2022) and therefore are promising targets for treatment. Impaired selective motor control also limits mobility and gait in predictable ways (Fowler et al., 2010) and warrants further research and development of more effective interventions.

This Research Topic collection examines the impact of neuromuscular impairments on gait and mobility and highlights promising areas of interventions.

In the study, “*Children with bilateral cerebral palsy use their hip joint to complete a step-up task*,” investigators Goyal et al. quantified differences in lower limb joint moment strategies of a step-up task, highlighting the impact of lower limb muscle weakness. They found differences in timing of lower limb joint moments during stance phase of a step-up task with increased dependence on the hip joint to keep the body upright and decreased use of the knee and ankle joints. The results identify the importance of hip extensor muscles in upright gait and posture and point to targeted methods of treatment to improve movement quality in bilateral CP.

“*A new methodological approach to characterize selective motor control in children with cerebral palsy*,” by Graci et al., offers a new clinical assessment of motor control in CP. The research identifies obligatory muscle co-activation patterns and mirror movements that correlate with reduced gross motor function and point to areas for targeted treatment.

The impacts on gait of the four neuromuscular impairments of spastic CP are described in “*Neuromuscular impairments of cerebral palsy: contributions to gait abnormalities and implications for treatment*,” by Clewes et al.. The article includes an easy-to-use clinical form for video-based gait evaluation to identify impacts of neuromuscular impairments at key points during the gait cycle, to better understand gait abnormalities and to target treatment that can improve gait abnormalities.

The article, “*Neuroanatomical correlates of gross manual dexterity in children with unilateral spastic cerebral palsy*,” by Beani et al., demonstrates a clinically useful MRI assessment and identifies important brain structure-function relationships that mediate upper limb function and manual dexterity. Findings suggest that timing of brain injury may correlate with presence of mirror movements and may account for variability in hand function.

Development of new innovative treatment is reported in the article, “*Development and evaluation of a BCI-neurofeedback system with real-time EEG detection and electrical stimulation assistance during motor attempt for neurorehabilitation of children with cerebral palsy*,” by Behboodi et al.. The article describes a novel Brain-Computer Interface (BCI) control of neuromuscular stimulation for ankle dorsiflexion to increase muscle strength, improve foot clearance during gait, and reduce risk of trips and falls.

The article, “*Pairing transcutaneous Vagus Nerve Stimulation (tVNS) with an intensive bimanual training in children and adolescents with cerebral palsy: study protocol of a randomized sham controlled trial*” by Oldrati et al., describes current methods to evaluate a promising treatment that pairs tVNS with bimanual training to improve upper limb function.

We hope that you enjoy this Research Topic collection and gain context for developing effective evaluations of etiology and neuromuscular impairments of spastic CP. We believe this context can help target treatment and improve mobility. We appreciate your interest and hope that you will be inspired to pursue these vital areas of research and development.
